# Effects of Monovalent Salt on Protein-Protein Interactions of Dilute and Concentrated Monoclonal Antibody Formulations

**DOI:** 10.3390/antib11020024

**Published:** 2022-03-31

**Authors:** Amy Y. Xu, Nicholas J. Clark, Joseph Pollastrini, Maribel Espinoza, Hyo-Jin Kim, Sekhar Kanapuram, Bruce Kerwin, Michael J. Treuheit, Susan Krueger, Arnold McAuley, Joseph E. Curtis

**Affiliations:** 1 NIST Center for Neutron Research, National Institute of Standards and Technology, 100 Bureau Drive, Mail Stop 6102, Gaithersburg, MD 20899, USA; amyxu@lsu.edu (A.Y.X.); nclark01@amgen.com (N.J.C.); susan.krueger@nist.gov (S.K.); 2 Institute for Bioscience and Biotechnology Research, University of Maryland, 9600 Gudelsky Drive, Rockville, MD 20850, USA; 3 Amgen Inc., One Amgen Center Drive, Thousand Oaks, CA 91320, USA; joey@amgen.com (J.P.); mespinoza27@ku.edu (M.E.); hkim@nektar.com (H.-J.K.); srkanapuram@yahoo.com (S.K.); bruce.kerwin@gmail.com (B.K.); treu805@gmail.com (M.J.T.); arnoldm@amgen.com (A.M.)

**Keywords:** protein-protein interactions, small-angle scattering, protein stability, formulation development

## Abstract

In this study, we used sodium chloride (NaCl) to extensively modulate non-specific protein-protein interactions (PPI) of a humanized anti-streptavidin monoclonal antibody class 2 molecule (ASA-IgG2). The changes in PPI with varying NaCl (*C_NaCl_*) and monoclonal antibody (mAb) concentration (*C_mAb_*) were assessed using the diffusion interaction parameter *k_D_* and second virial coefficient *B*_22_ measured from solutions with low to moderate *C_mAb_*. The effective structure factor *S(q)_eff_* measured from concentrated mAb solutions using small-angle X-ray and neutron scattering (SAXS/SANS) was also used to characterize the PPI. Our results found that the nature of net PPI changed not only with *C_NaCl_*, but also with increasing *C_mAb_*. As a result, parameters measured from dilute and concentrated mAb samples could lead to different predictions on the stability of mAb formulations. We also compared experimentally determined viscosity results with those predicted from interaction parameters, including *k_D_* and *S(q)_eff_*. The lack of a clear correlation between interaction parameters and measured viscosity values indicates that the relationship between viscosity and PPI is concentration-dependent. Collectively, the behavior of flexible mAb molecules in concentrated solutions may not be correctly predicted using models where proteins are considered to be uniform colloid particles defined by parameters derived from low *C_mAb_*.

## 1. Introduction

Monoclonal antibodies (mAbs) are a major class of protein biotherapeutics widely used to treat a variety of diseases [1]. Owing to gastric degradation and their inherent high molecular weights, the most preferable administration route for mAb therapeutics is through subcutaneous (SC) injections [2,3]. Due to their relatively low specificity and restrictions on the injection volume for SC administration, mAbs are frequently formulated at high concentrations to achieve desired therapeutic dosages [4,5,6]. The spatial distances between individual mAb molecules decrease significantly with increasing protein concentration, leading to self-association and elevated solution viscosity [7,8,9]. To this end, excipients are frequently included in solution formulations to improve the stability, bioavailability and manufacturability of mAb products [10,11,12,13].

Although it is undesirable to have oligomers in mAb products, small fractions of such higher-order structures are present in mAb formulations [14,15,16]. The formation of oligomers initiates when two mAb molecules form dimers. The self-association between two mAb molecules can be represented as M+M ⇌D, where *M* and *D* represent the monomer and dimer concentration at equilibrium, respectively. The equilibrium constant *K* is expressed as [*D*]/[*M*]^2^; thus, it describes the tendency of mAb monomers to form dimers. Since *K* remains constant, an increase in the monomer concentration will lead to an increase in the number of dimers. On the other hand, removing dimers from the system will stimulate the formation of more dimers until the monomer-dimer equilibrium is restored. Therefore, from the basis of chemical equilibrium, the formation of higher-order structures is inevitable, and the number of dimers present in the system is dictated by the value of *K*. Numerous studies have shown that the percentage of dimers could vary between 0.9% to 2.8% in certain mAb formulations [17,18,19]. Moreover, Chaturvedi et.al. measured the self-association of several mAbs and found the association constants covered a wide range from 10 µM to 1 mM for weakly associative mAbs [20]. In general, the formation of mAb oligomers can be expressed as:

M+M⇌D+M⇌T+M⇌⋯⇌O

where *M*, *D*, *T* and *O* represent the monomer, dimer, trimer and oligomer concentrations, respectively. Each step has its own equilibrium constant that can be calculated by the concentration of products and reactants at equilibrium. As mentioned earlier, excipients such as salts and sugars are often included in the formulation to improve the stability of mAb molecules by reducing the extent of self-association [11]. It is known that the monomer-dimer equilibrium can change from one solution environment to another by, for example, adding small molecule inhibitors [21], salts [22] and sugars [23]. Therefore, the presence of excipients in a solution can alter oligomer formation by altering various aspects that contribute to the native equilibrium constants.

Both experimental and computer-based approaches have been developed to aid the formulation development of mAb products. For computer simulations, different models have been developed to capture various molecular features of mAbs for improved predictions of PPI. Among others, coarse-grained bead models that account for the shape and surface anisotropy of mAb molecules have been used to predict the stability and viscosity of concentrated mAb formulations [24,25,26]. While advancements have been made in computer simulations, experimental data are needed to validate the force field used for the simulation and select molecular features that best describe a particular mAb molecule.

In recent studies of the self-association of proteins, protein molecules have been modelled as colloidal particles [27,28]. In this formalism, the interaction between two protein molecules is governed by steric repulsion. Protein molecules can also interact due to surface anisotropy and solvent-mediated interactions [29]. These interactions can be either attractive or repulsive in nature [29,30]; therefore, they diminish or enhance the interaction between two protein molecules. When considering proteins as colloid particles, the tendency of proteins to stay in their monomeric form is typically referred to as their colloidal stability, and it is dominated by the net balance between repulsive and attractive protein-protein interactions (PPI) [31].

A wide range of characterization methods has been developed to predict the colloidal stability of mAbs in various formulations. These techniques are used to extract experimental parameters containing information on the net PPI, with the underlying assumption that mAbs are colloidally stable if the net PPIs are repulsive and unstable if the net PPIs are attractive [32,33,34]. Dynamic light scattering (DLS) and static light scattering (SLS) are two widely used techniques to study the colloidal stability of protein therapeutics in solution [35,36]. The diffusion interaction parameter *k_D_* and the second virial coefficient *B*_22_ are estimated from DLS and SLS, respectively. Although *k_D_* and *B*_22_ are measures of the deviation from non-ideal solution properties which obey the Van’t Hoff relation, they have been used to predict the PPI and viscosity for concentrated mAb formulations [37,38,39,40,41,42,43]. In addition to DLS/SLS, the effective structure factor *S(q)_eff_* measured from small-angle X-ray and neutron scattering (SAXS/SANS) also provides information on spatial arrangements and intermolecular interactions of mAbs in solution. In recent years, there have been a number of studies of PPI using SAXS/SANS [24,44,45,46,47,48,49]. Our previous study demonstrated the use of SAXS for studying the stability and viscosity of mAbs under various formulation conditions [11]. One of the major differences between *k_D_*/*B*_22_ and *S(q)_eff_* is that the latter can be measured directly from concentrated mAb solutions up to hundreds of mg/mL. Thus, *S(q)_eff_* provides a direct probe of PPI present in concentrated formulations. Previously, we examined the correlations between *k_D_*/*B*_22_ and *S(q)_eff_* measured from commonly used excipient formulations, where excipients were formulated at particular concentrations, for example, 300 mM sucrose and 200 mM glycine [11]. In this study, we focus on only one excipient: NaCl. The thermodynamic, hydrodynamic and structure of a human anti-streptavidin monoclonal antibody (ASA-IgG2) was characterized with various amounts of NaCl in solution, ranging from 0 mmol/L (mM) up to 1200 mM. High NaCl concentrations were included to extensively modulate the PPI and monomer-oligomer equilibrium of ASA-IgG2, allowing us to thoroughly characterize the effects of NaCl on interactions among concentrated mAb molecules. In addition, our study includes NaCl concentrations greater than 150 mM, as high concentrations of salt are extensively used in protein precipitation [50,51]; thus, knowing the changes in PPI with an increasing amount of salt will allow a fundamental understanding of the physics of PPI as probed by the various scalar and derived scattering parameters determined in this study.

## 2. Materials and Methods

### 2.1. Protein Preparation

Stock solution of anti-streptavidin monoclonal immunoglobulin antibody class 2 (ASA-IgG2) was taken from a −80 °C freezer and thawed at room temperature. The mAb solutions were dialyzed against 10 mM sodium acetate buffer at pH 5.2 overnight at 4 °C for complete buffer exchange. For SANS measurements, a second dialysis step was included, in which the ASA-IgG2 solution was dialyzed against 10 mM sodium acetate buffer prepared using D2O. The pD of the deuterated buffer was adjusted to 5.2 with 10 M NaOD. The unit M stands for the molar concentration of mol/L, whereas mM stands for the concentration of 10^−3^ mol/L. After dialysis, ASA-IgG2 in desired buffer conditions were concentrated to 215 mg/mL using an Amicon centrifugal concentrator with a molecular weight cut-off (MWCO) of 3 kDa. In order to prevent unwanted protein gelation, extra precautions were taken during the concentration step. For example, the mAb solutions were only allowed to spin at 4000× *g* at 18 °C for a maximum of 10 min at a time. After each centrifugation step, the samples were gently but thoroughly mixed before the next cycle. The concentrated solutions were diluted to desired protein concentrations using appropriate amounts of dialysis buffer or 5 M NaCl solution or both. Certain samples with high mAb and NaCl concentrations could not be made due to difficulties in preparing concentrated ASA-IgG2 (greater than 215 mg/mL) and NaCl (greater than 5 M) stock solutions. The concentration of ASA-IgG2 mAb was determined from its absorbance at 280 nm with a percent extinction coefficient of 16 [52]. Samples were thoroughly mixed by gentle pipetting and spun at 16,000× *g* for 15 min prior to SAXS/SANS measurements.

Certain commercial equipment, instruments, or materials (or suppliers, or software, …) are identified in this paper to foster understanding. Such identification does not imply recommendation or endorsement by the National Institute of Standards and Technology, nor does it imply that the materials or equipment identified are necessarily the best available for the purpose.

### 2.2. Small-Angle X-ray Scattering (SAXS)

SAXS measurements were performed on the BioSAXS beamline at the Cornell High-Energy Synchrotron Source (CHESS) in Ithaca, NY, USA. Samples were centrifuged at 13,500× *g* for 30 min and then transferred to a 96-well plate. The plate was placed on a robotic platform, allowing samples to be automatically loaded into a capillary sample cell for X-ray exposures [53,54]. Scattering profiles with the *q*-range of 0.008–0.8 Å^−1^ were recorded with an X-ray energy of 9.88 keV at 25 °C. A total of 32 × 5-s exposures were taken from each sample with oscillations in order to limit radiation damage. Between each measurement, the capillary sample cell was washed thoroughly with three capillary volumes (CVs) of ultrapure water, 5 CVs of 10% Hellmanex in 20% ethanol, 4 CVs of 70% ethanol, 4 CVs of ultrapure water, and, finally, 2 CVs of buffer. Buffer was measured between protein samples to ensure the absence of contaminants in the capillary sample cell. SAXS data were reduced and processed using the *BioXTAS RAW* software [55] to produce scattering intensity *I(q)* vs. *q* scattering profiles, where *q* is the scattering vector and is defined as 4π sin(*θ*)/*λ*, where *λ* is the wavelength and 2*θ* is the scattering angle.

### 2.3. Small-Angle Neutron Scattering (SANS)

SANS profiles were measured on the Center for High-Resolution Scattering (CHRNS) 30-m SANS instrument (NGB30) at the NIST Center for Neutron Research (NCNR), National Institute of Standards and Technology, Gaithersburg, MD, USA. Samples were prepared in a deuterated buffer with varying amounts of NaCl and mAbs. Sample solutions were loaded into quartz cells with path lengths of either 1 mm or 2 mm for measurements. In particular, the 2 mm cells were used for samples with *C_mAb_* less than 50 mg/mL, while 1 mm cells were used for more concentrated samples. All measurements were made at 25 °C. A total of three different sample-to-detector distances were used (1.5 m, 5.0 m, and 13.0 m) to cover a *q*-range from 0.007 Å^−1^ to 0.3 Å^−1^. SANS data reduction and model fitting were performed using NCNR SANS reduction and analysis macros in the IgorPro software package [56] to produce *I(q)* vs. *q* scattering profiles that are corrected for scattering from the buffer as well as incoherent scattering from hydrogen atoms in the sample.

### 2.4. Calculation and Analysis of Effective Structure Factor S(q)_eff_

For an ideal system that consists of monodisperse, homogeneous and isotropic dispersions of spherical particles, the total scattering *I(q)* can be expressed as:

(1) $$I\left(q\right)=\frac{N}{V}\;{\left(\Delta \rho \right)}^{2}V_{p}^{2}P\left(q\right)S\left(q\right)$$

where (*N*/*V*) and *V_p_* represent the number density and volume of scattering particles, respectively, and ∆*ρ* represents the difference in scattering length densities between the particle and solvent background. *P*(*q*) is the form factor that is related to the size and shape of the scatterers, whereas the structure factor, *S(q)*, is related to the spatial arrangements of particles and thus contains information on the interparticle interactions. Since mAbs are non-spherical and anisotropic, the experimentally determined *S(q)* is often referred to as the effective structure factor *S(q)_eff_* since it is affected by the shape and anisotropy of interactions between molecules [57,58]. The effective structure factor *S(q)_eff_* is concentration-dependent, and it can be determined from the total scattering *I(q)* using the following equation:

(2) $$S{\left(q\right)}_{eff}=\frac{I{\left(q\right)}_{concentrated}}{s\times I{\left(q\right)}_{dilute}}$$

where *I(q)_dilute_* is the scattering profile measured from dilute solutions where only *P*(*q*) contributes towards the total scattering. In this study, *I(q)* measured from 2 mg/mL ASA-IgG2 solutions were considered as *I(q)_dilute_* since *S(q)* is equal to 1 at low concentration. *I(q)_concentrated_* are the scattering profiles measured from concentrated solutions, where not only the *P*(*q*) but also the *S(q)* contribute toward the total scattering. The value *s* is the scaling factor for the given concentration where the scattering was measured and is used to normalize the scattering profiles measured from various concentrations [59,60].

Experimentally, the concentration of ASA-IgG2 antibody was measured before scattering experiments so that the scaling factor *s* could be calculated based on this information. The *S(q)_eff_* profiles were obtained from the measured *I(q)_concentrated_* and *I(q)_dilute_* scattering profiles, as shown in Equation (2). Special attention was paid before dividing *I(q)_concentrated_* with *s*×*I(q)_dilute_* to make sure that the scattering profiles in the high-*q* region (for example, the linear region from 0.10 Å^−1^ to 0.14 Å^−1^) were normalized as shown in Supporting Information Supplementary Figure S1. Since only *P*(*q*) contributes to *I(q)* in the high-*q* region, the scattering profiles should be identical. Therefore, checking the high-*q* scattering profiles ensured the proper extraction of structure factors; detailed instructions on how to extract *S(q)_eff_* profiles can be found in previous publications [11,60]. Our previous study demonstrated that flexible mAbs can be treated as spheres at a larger length scale where configurational variations of mAb molecules do not perturb interparticle correlations [61]. Therefore, *S(q)_eff_* profiles can be fitted using statistical mechanical models of the structure factor [62]. These include: (1) the hard sphere model, where the steric repulsion is considered to be the only intermolecular interaction; (2) the Hayter–Penfold model, where additional Coulomb repulsions between molecules are also considered; and (3) the Two–Yukawa model, where both attractive and repulsive interactions are taken into account [11,61,63,64,65]. The *S(q)_eff_* were analyzed using the Orstein–Zernike (OZ) integral equation with the assumption that the protein molecules were spherical particles. In the hard sphere model, Percus−Yevick (PY) closure was used for the calculation of *S(q)_eff_*, and the interparticle potential *U*(*r*) was expressed as [66]:

(3) $$U\left(r\right)=\{\begin{array}{c} \infty ,\;\;r<2R\\ 0,\;\;r\geq 2R \end{array}$$

where *r* is the center of mass separation between two spheres with a radius of *R*. In the Hayter–Penfold model, the interaction potential *U*(*r*) was expressed as [65]:

(4) $$U\left(r\right)=\{\begin{array}{c} \infty ,\;\;r<\delta \\ \frac{Z^{2}}{\pi \epsilon_{0}\epsilon {\left(2+\kappa \delta \right)}^{2}}\frac{e^{-\kappa \left(r-\delta \right)}}{r},\;\;r\geq \delta \end{array}$$

where *Z* and *δ* are the effective charge and diameter of the particle, respectively, *κ* is the inverse of the Debye–Huckel screening length, $\epsilon_{0}$ is the permittivity of free vacuum, and ϵ is the dielectric constant of the solvent [67]. In the Two–Yukawa model, the reduced interaction potential *U*(*x*) was expressed as [68]:

(5) $$U\left(x\right)=\{\begin{array}{c} \infty ,\;\;x<1\\ -K_{1}\frac{e^{-Z_{1}\left(x-1\right)}}{x}-K_{2}\frac{e^{-Z_{2}\left(x-1\right)}}{x},\;\;x\geq 1 \end{array}$$

where *x* is equal to *r*/*δ*. *K*_1_ and *Z*_1_ are the strength and range of attractive interactions, whereas *K*_2_ and *Z*_2_ are the strength and range of repulsive interactions, respectively. *S(q)_eff_* is represented by *S*(0)*_eff_*, the value of *S(q)_eff_* extrapolated to *q* = 0, which was obtained by fitting the *S(q)_eff_* profiles to the above models.

### 2.5. Dynamic Light Scattering (DLS)

DLS experiments were performed to obtain the diffusion interaction parameter *k_D_* for ASA-IgG2 prepared in various NaCl solutions at 25 °C. Samples were centrifuged at 16,000 rpm for 5 min prior to measurements. Aliquots of 120 µL were loaded onto a 96-well plate (SensoPlates by Greiner Bio-One Inc., Charlotte, NC, USA) and measured on a DynaPro II plate-reader (Wyatt Technologies, Santa Barbara, CA, USA) with a laser wavelength of 830 nm. For each NaCl concentration, the mutual diffusion coefficient *D_m_* was measured from 1 series of mAb solutions with *C_mAb_* ranging from 1 mg/mL to 10 mg/mL. The value of *k_D_* was determined as the linear slope of *D_m_* versus *C_mAb_*:

(6) $$D_{m}=D_{0}\left(1+k_{D}C_{mAb}\right)$$

where *D*_0_ is the self-diffusion coefficient of the protein and can be determined from the intercept of plot.

### 2.6. Composition Gradient Multi-Angle Light Scattering (CG-MALS)

CG-MALS is a technique that combines equilibrium experimental data with interpretation facilitated by fitting to a developing set of theoretical models. In this study, CG-MALS experiments were performed under room temperature on a Calypso composition gradient system (Wyatt Technology, Santa Barbara, CA, USA) in conjunction with a DAWN-HELEOS MALS photometer, an Optilab rEX on-line differential refractometer for concentration measurements (all detectors from Wyatt Technology), as well as offline UV measurements by Nanodrop. The photometer was calibrated with toluene. ASA-IgG2 stock solutions were prepared in 10 mM sodium acetate buffer at pH5.2 with various NaCl concentrations. The stock solutions were then manually diluted with appropriate buffers to yield secondary solutions of 50 mg/mL. The secondary solutions were purified through 0.22 µm Anotop ceramic filters (Whatman GE, Billerica, MA, USA) before the measurements. The DAWN laser power was reduced to avoid detector saturation. For each CG-MALS measurement, both sample and buffer were combined in the auto-diluter to produce concentration series down to 5 mg/mL, injected (1 mL total) into the flow cells at 1 mL/min, and the flow was stopped for 300 s to acquire data after complete equilibration in the DAWN and Optilab flow cells before creating and injecting a subsequent concentration. One dilution experiment was performed for each NaCl concentration. Instrument control, data acquisition and data analysis were all carried out with the Calypso software, which implements the protein interaction model fitting, including a single-species effective hard-sphere volume approximation (EHSVA) based on Equations (7)–(10) [69,70]:

(7) $$c_{tot}=c_{1}+\underset{i>1}{\sum }ic_{i}$$

(8) $$c_{i}=K_{i}{\left(c_{1}\right)}^{i}$$

(9) $$R\left(0\right)=\tilde{K}\;{\left(\frac{dn}{dw}\right)}^{2}\left[M^{2}c_{1}+\underset{i>1}{\sum }{\left(iM\right)}^{2}c_{i}\right]$$

(10) $$\frac{R\left(0\right)}{\tilde{K}}={\left(\frac{d_{n}}{d_{w}}\right)}^{2}\frac{M_{w1}+M\sum_{i>1}iw_{i}}{1+8vw_{tot}+30{\left(vw_{tot}\right)}^{2}+73.4{\left(vw_{tot}\right)}^{3}+\frac{141.2{\left(vw_{tot}\right)}^{4}}{1-1.368vw_{tot}}}$$

where *w_tot_* represents the combined mass/volume concentration of the monomers and all oligomers, *v* is the specific volume, *c_tot_* is the total molar concentration of free and bound protein monomers in solution; and $c_{1}$ and $c_{i}$ are the partial concentrations of free monomers and oligomers, respectively. *K_i_* is the equilibrium association constant for the monomer-oligomer association. *R*(0) is the Rayleigh ratio determined from the intensity of scattered light over multiple scattering angles. $\tilde{K}$ is calculated from the free-space scattering wavelength λ_0_ and solvent refractive index *n*_0_ as (π n_0_ [2]/N_A_λ_0_ [4]). *M* is the monomer molar mass; *dn*/*dw* is the refractive index increment of proteins in solution with respect to weight concentration *w*. This approach describes the light scattering from non-ideal, self-associating proteins in terms of the monomeric molar mass and a single quantity representing the repulsive component of thermodynamic nonideality.

### 2.7. Viscosity Measurement

Viscosity experiments were performed on an ARG2 cone and plate rheometer (TA Instruments) using a 20 mm 1.988° cone plate equipped with a steel Peltier plate (TA part # 511206.905). Antibody solutions were equilibrated to room temperature prior to viscosity measurements. A sample load volume of 80 µL and a temperature set point of 23 °C were determined to be optimal for the purpose of this study. A shear sweep was performed for a shear range of 10 to 1000 s^−1^. Solution viscosity measured at 1000 s^−1^ was used to illustrate the viscosity of samples varying in NaCl and mAb concentrations.

## 3. Results and Discussions

### 3.1. Effects of NaCl Concentration on PPI: A Comparative Study between k_D_/B_*22*_ and S(q)_eff_

**Figure 1:**
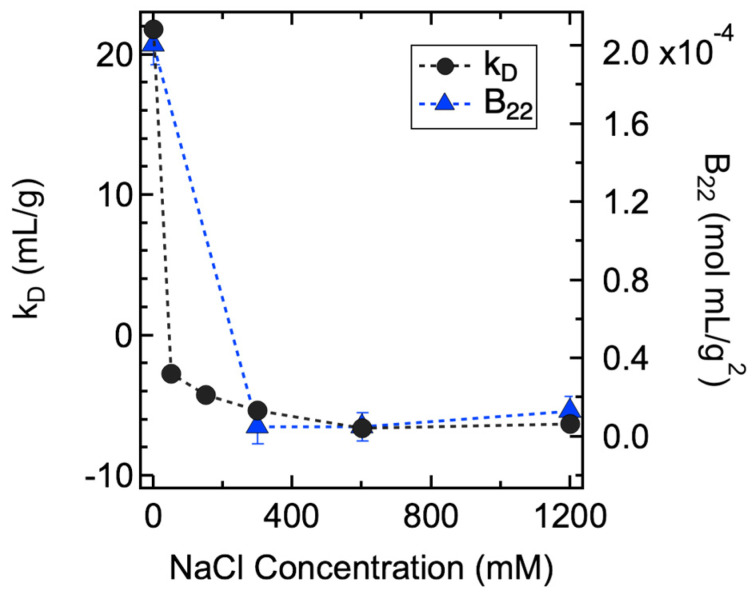
Summary of *k_D_* (black circle) and *B*_22_ (blue triangle) values measured at various NaCl concentrations. Error bars represent the standard errors; some errors are smaller than the plotting symbols. In particular, uncertainties in *k_D_* were determined as the propagation of standard errors of the coefficients from the linear regression.

In this study, both *k_D_*/*B*_22_ and *S(q)_eff_* were used to characterize the PPI of ASA-IgG2 molecules in solution. In particular, *k_D_* values were measured by DLS on solutions with *C_mAb_* less than 10 mg/mL, whereas *B*_22_ values were obtained by fitting the CG-MALS results collected from 50 mg/mL mAb solutions, both while varying *C_NaCl_*. As shown in Figure 1, the *k_D_* values decreased significantly from 21.8 mL/g to −2.8 mL/g with the initial addition of 50 mM NaCl. Further increase in *C_NaCl_* led to a relatively small decrease in *k_D_* values until a plateau of −6.5 mL/g was reached. A similar trend was observed for *B*_22_ values across the examined NaCl concentration range. The observed changes in *k_D_* and *B*_22_ values implied that the PPI between mAb molecules were strongly repulsive without NaCl. Increasing NaCl concentration resulted in the screening of surface charges on mAb molecules by Na^+^ and Cl^−^ ions; hence, smaller *k_D_* and *B*_22_ values were measured due to decreased electrostatic repulsions. Further increases in *C_NaCl_* above 600 mM caused negligible changes in the net PPI, as was evident from the constant *k_D_* and *B*_22_ values. While *B*_22_ is a measure of the strength of pairwise intermolecular interactions, *k_D_* is a result of both thermodynamic and hydrodynamic parameters. Thus, unlike *B*_22_, *k_D_* does not define attractive or repulsive PPI with values below or above zero. It has been reported that a better *k_D_* value for determining the nature of net PPI is around −8 mL/g for mAbs, meaning that a result above this cut-off value is indicative of net repulsive PPI and vice versa [71,72]. Therefore, the *k_D_* and *B*_22_ values measured from all samples were greater than the cut-off values, suggesting the net PPI were repulsive among all examined NaCl concentrations.

**Figure 2:**
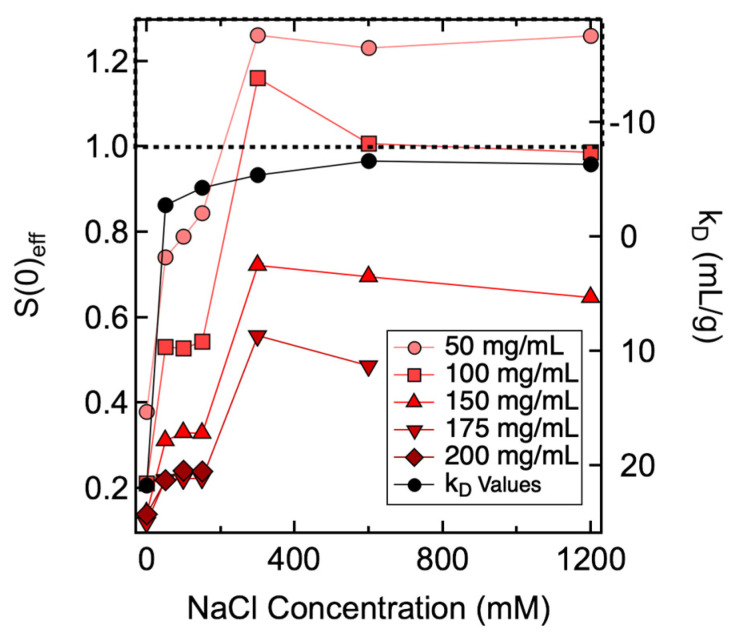
Changes in *S*(0)*_eff_* (left axis) and *k_D_* (right axis) values as a function of NaCl concentration. The dotted line represents cut-off values of *S*(0)*_eff_* of 1 and *k_D_* of −8 mL/g. Divided by the dotted line, the top and bottom panel of the figure highlight samples with *S*(0)*_eff_* and *k_D_* values that are indicative of attractive and repulsive PPI, respectively. Lines between data points are added to guide the eye. Uncertainties in *S*(0)*_eff_* represent the standard error of the mean for three different structure factor fits; some error bars are smaller than the plotting symbols in this figure. Uncertainties in *k_D_* were determined as the propagation of standard errors of the coefficients from the linear regression.

The SAXS and SANS profiles measured from ASA-IgG2 solutions prepared with varying *C_mAb_* and *C_NaCl_* are shown in Supplementary Figures S2 and S3 from Supporting Information. No power-law scattering was observed in the low-*q* region, suggesting no long-lived, large-scale structures were formed in all samples examined. The *S(q)_eff_* were used to characterize the PPI among ASA-IgG2 molecules as a function of *C_mAb_* and *C_NaCl_*. The magnitude of *S(q)_eff_* was quantified by *S*(0)*_eff_* values as *q* approached zero (see Supplementary Figure S4 for experimental and fitted *S(q)_eff_* profiles). Values of *S*(0)*_eff_* were obtained by fitting the *S(q)_eff_* profiles using appropriate models and used to determine the nature of net PPI: a value greater than 1 indicates the net PPI are attractive, whereas a value less than 1 suggests the net PPI are repulsive. Since *k_D_* and *B*_22_ values were in close agreement, we used *k_D_* results to represent dilute solution properties and compared them with *S*(0)*_eff_* values measured from solutions with various mAb and NaCl concentrations (Figure 2).

**Figure 3:**
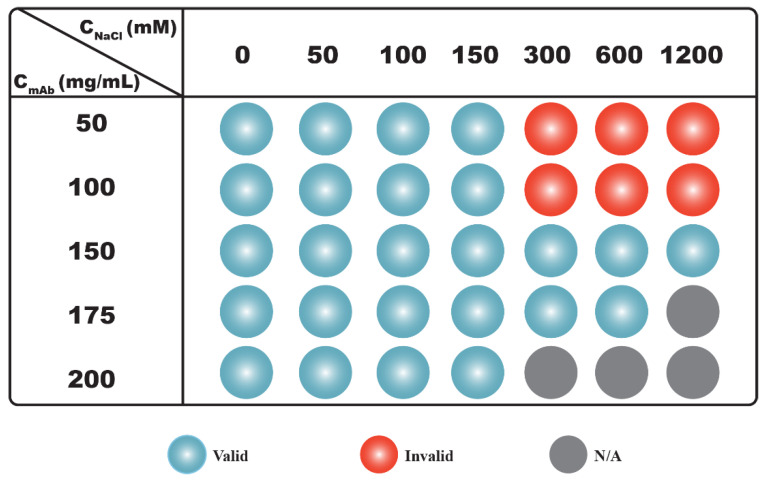
Correlations between *k_D_* and *S*(0)*_eff_* values. Conditions under which *k_D_* is greater than −8 mL/g and *S*(0)*_eff_* value is less than 1 are represented with blue spheres. Under these conditions, both parameters suggest the net PPI are repulsive. Conditions under which *k_D_* is greater than −8 mL/g, but *S*(0)*_eff_* is greater than 1 are represented with red spheres. Under these conditions, *k_D_* values suggest net PPI are repulsive, while *S*(0)*_eff_* values suggest the net PPI are dominated by attractions. Experimental conditions under which mAb samples are not prepared/measured are shown in grey.

Results shown in Figure 2 demonstrate that the net PPI was not only dependent on *C_NaCl_*, but it also varied with *C_mAb_*. The *S*(0)*_eff_* results can be clarified if we divide the results into two regimes along the axis of *C_NaCl_* (Figure 3). When *C_NaCl_* was below 300 mM, *S*(0)*_eff_* values measured from all mAb concentrations were less than 1, indicating that the net PPI were mainly repulsive. Hence, *S*(0)*_eff_* agreed with *k_D_* results when *C_NaCl_* was less than 300 mM, since both showed net repulsive PPI. As *C_NaCl_* increased to ≥300 mM, *S*(0)*_eff_* values changed with increasing mAb concentrations: *S*(0)*_eff_* values measured from 50 mg/mL mAb solutions were all greater than 1, suggesting the net PPI were attractive. For 100 mg/mL samples, the *S*(0)*_eff_* value changed from greater to almost equal to 1 as *C_NaCl_* increased from 300 mM to 1200 mM, suggesting attractive forces were balanced by repulsive forces with increasing *C_NaCl_*. As *C_mAb_* further increased to ≥150 mg/mL, the net PPI became repulsive, as evident by *S*(0)*_eff_* values less than 1. Therefore, as illustrated in Figure 3, correlations between *S*(0)*_eff_* and *k_D_* were only valid for samples prepared with low to intermediate *C_NaCl_*, i.e., up to 150 mM for the current study. The correlation became unreliable when *C_NaCl_* or the ionic strength of the solution was higher. Under such buffer conditions, the nature of net PPI changed with *C_mAb_*.

**Figure 4:**
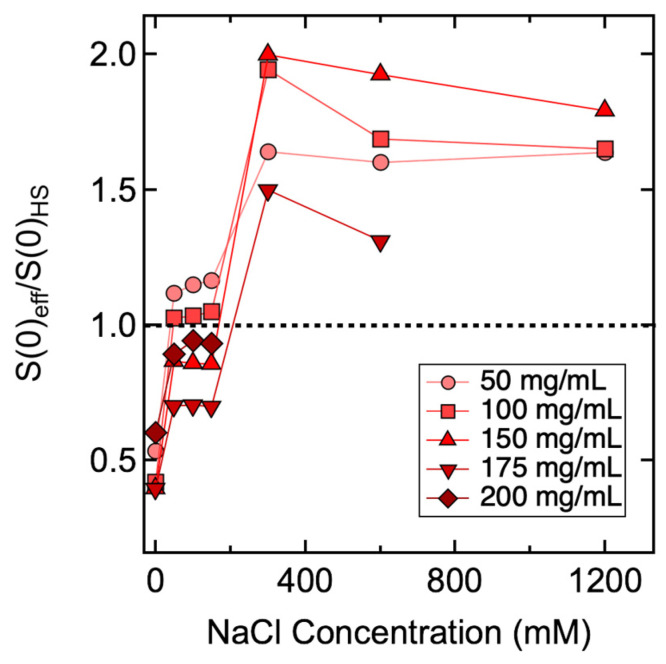
Summary of *S*(0)*_eff_*/*S*(0)*_HS_* ratios measured from ASA-IgG2 antibody solutions with varying mAb and NaCl concentrations. The dotted line represents a cut-off value of 1. Lines between data points are added to guide the eye. A ratio less than 1 implies the presence of repulsive interactions in addition to steric repulsion, whereas a ratio greater than 1 implies the presence of both repulsive and attractive interactions. Uncertainties in this plot are propagated from the standard errors in *S*(0)*_eff_* values; error bars are smaller than the plotting symbols in this figure.

A hard-sphere model is considered the most basic fitting model for *S(q)_eff_*. Other commonly used fitting models are built with consideration of additional intermolecular interactions. Therefore, different types of intermolecular interactions that together contribute to the net PPI are revealed through the fitting of *S(q)_eff_*. To this end, *S*(0)*_eff_* values were compared with theoretical *S*(0)*_HS_* values calculated from a hard-sphere model with PY closure. If *S*(0)*_eff_* value was less than *S*(0)*_HS_*, additional repulsive interactions other than steric repulsion were present, in which case the Hayter–Penfold model was used to fit the *S(q)_eff_* profile. If the *S*(0)*_eff_* value was greater than *S*(0)*_HS_*, the Two–Yukawa model was used to fit the *S(q)_eff_* profile by considering both repulsive and attractive interactions [63]. The ratios between *S*(0)*_eff_* and *S*(0)*_HS_* values were summarized and presented in Figure 4 to provide more insights into the mixture of forces that together contribute towards the net PPI.

**Figure 5:**
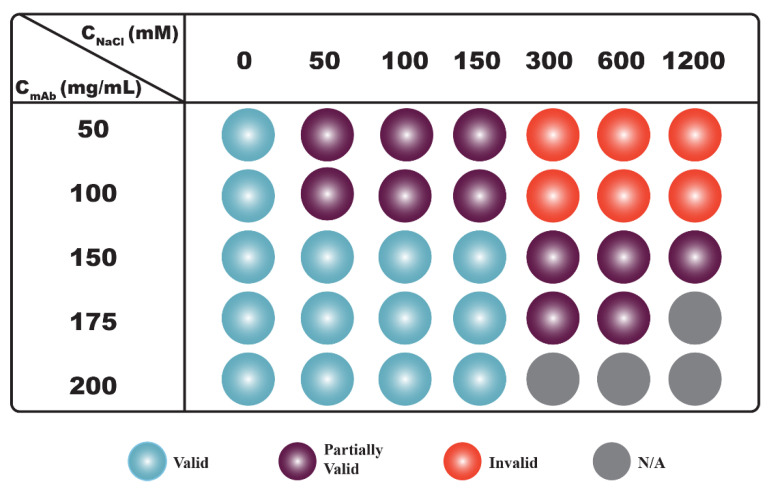
Correlations between *k_D_*, *S*(0)*_eff_* and *S*(0)*_eff_*/*S*(0)*_HS_* ratios. Conditions under which *k_D_* is greater than −8 mL/g and *S*(0)*_eff_* as well as *S*(0)*_eff_*/*S*(0)*_HS_* values are less than 1 are represented with blue spheres. Under these conditions, all three parameters suggest the net PPI are repulsive. Conditions under which *k_D_* is greater than −8 mL/g, but *S*(0)*_eff_* as well as *S*(0)*_eff_*/*S*(0)*_HS_* values are greater than 1 are represented with red spheres. Under these conditions, *k_D_* values suggest net PPI are repulsive, but *S*(0)*_eff_* values suggest the net PPI are dominated by attractions. Conditions under which *k_D_* is greater than −8 mL/g, *S*(0)*_eff_* is less than 1, but *S*(0)*_eff_*/*S*(0)*_HS_* is greater than 1 are represented with purple spheres. Under these conditions, both *k_D_* and *S*(0)*_eff_* suggest the net PPI are repulsive; however, the *S*(0)*_eff_*/*S*(0)*_HS_* ratio suggests the presence of non-dominating attractive interactions. Experimental conditions under which mAb samples are not prepared/measured are shown in grey.

It can be seen from Figure 4 that some samples demonstrated *S*(0)*_eff_*/*S*(0)*_HS_* values greater than 1, while their corresponding *S*(0)*_eff_* values were less than 1, implying attractive interactions were present among mAb molecules despite the net PPI being repulsive. An examination of correlations among *k_D_*, *S*(0)*_eff_* and *S*(0)*_eff_*/*S*(0)*_HS_* results are shown in Figure 5. It was found that *k_D_* values were in qualitative agreement with *S*(0)*_eff_* results for samples prepared without added NaCl; both suggested the net PPI were dominated by repulsions (blue spheres in column 1 in Figure 5). Moreover, the *S*(0)*_eff_*/*S*(0)*_HS_* values measured from these samples were also less than 1, implying the net PPI were mainly attributed to steric and electrostatic repulsion. For samples prepared with *C_NaCl_* up to 150 mM, the correlation between *k_D_* and *S*(0)*_eff_* persisted since both were below the cut-off values between net repulsive and attractive interactions, suggesting the net PPI were dominated by repulsion (blue and purple spheres in columns 2–4 in Figure 5). However, *S*(0)*_eff_*/*S*(0)*_HS_* values measured from samples prepared with a *C_mAb_* less than 150 mg/mL were greater than 1 (purple spheres in rows 2–4 of Figure 5), suggesting the presence of attractive interactions despite the net PPI being repulsive. Interestingly, the *S*(0)*_eff_*/*S*(0)*_HS_* values became less than 1 when *C_mAb_* increased to ≥150 mg/mL (blue spheres in rows 3–5 in Figure 5), suggesting attractive interactions were significantly reduced and that the net PPI were dominated by repulsions again as mAb molecules became more concentrated in solution. As discussed earlier, correlations between *k_D_* and *S*(0)*_eff_* became unreliable as *C_NaCl_* increased to ≥300 mM since the *S*(0)*_eff_* values demonstrated a monotonic change across 1 with varying *C_mAb_*. The *S*(0)*_eff_* values measured from 50 mg/mL and 100 mg/mL mAb solutions were greater than 1, suggesting net PPI were attractive, thus contradicting the predicted PPI based on *k_D_* values (red spheres in rows 1–2 in Figure 5). When *C_mAb_* increased to 150 mg/mL and above, although the *S*(0)*_eff_* values were less than 1, the *S*(0)*_eff_*/*S*(0)*_HS_* values measured from these samples were all well above 1, implying the presence of non-dominating attractive interactions even though the net PPI were repulsive (purple spheres in rows 3–4 in Figure 5).

Therefore, experimental results suggested the correlation between *k_D_*/*B*_22_ and *S(q)_eff_* was the strongest only without added NaCl, where the net PPI were mainly dominated by steric and electrostatic repulsion. With the addition of NaCl, attractive forces were found to arise among mAb molecules, and correlations between *k_D_*/*B*_22_ and *S(q)_eff_* were found to vary with *C_mAb_* as well. This finding highlights the complex nature of PPI and the fact that the dilute solution measurements may not correctly reflect changes in PPI at higher *C_mAb_*.

### 3.2. Composition Gradient Multi-Angle Light Scattering (CG-MALS) Results Revealed the Presence of Higher-Order Structures

The ideal formulation for mAbs should ensure the net PPI are dominated by repulsive forces among all relevant mAb concentrations [36]. Detailed analysis of *S(q)_eff_* revealed that although the net PPI appeared to be repulsive under certain experimental conditions (a combination of both *C_NaCl_* and *C_mAb_*), attractions could also be present among mAb molecules. In order to provide direct evidence of the existence of attractive interactions, CG-MALS measurements were performed to examine the formation of transient, higher-order structures in solution. Thus, 50 mg/mL ASA-IgG2 mAb solutions prepared with various *C_NaCl_* were subjected to CG-MALS measurements since correlations between *k_D_*, *S*(0)*_eff_* and *S*(0)*_eff_*/*S*(0)*_HS_* turned from valid to invalid with increasing *C_NaCl_* at this *C_mAb_*.

**Figure 6:**
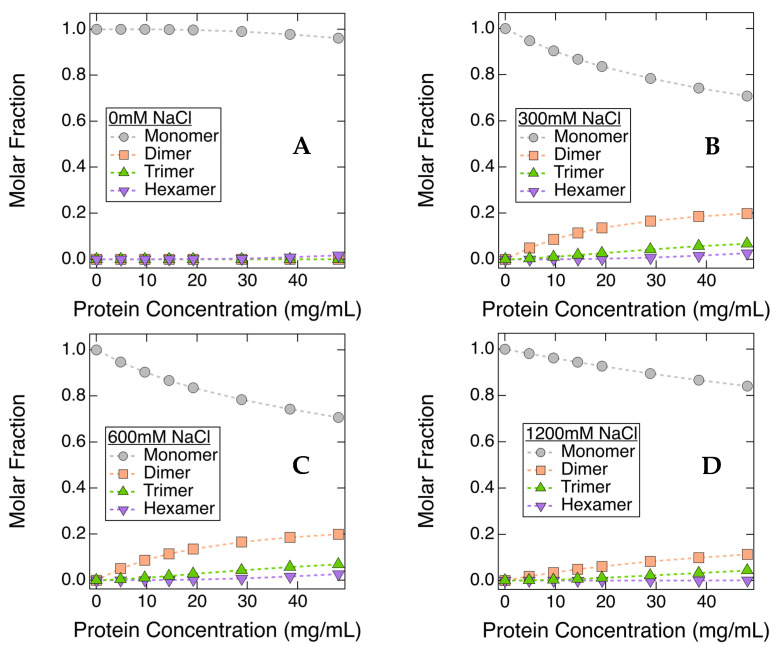
Molar fractions of ASA-IgG2 monomer and other self-association products in various NaCl solutions: 0mM NaCl (**A**), 300 mM NaCl (**B**), 600 mM NaCl (**C**), and 1200 mM NaCl (**D**). Although smaller than the plotting symbols, error bars represent standard errors.

The effects of NaCl on the degree of protein self-association are represented in Figure 6. The molar fractions of various species were estimated by fitting the light scattering profiles measured at each mAb concentration [36,73]. Higher-order oligomeric states included in the fits were dimers, trimers and hexamers. In a solution where no NaCl was added, the molar fraction of ASA-IgG2 monomer decreased only slightly with increasing *C_mAb_*, implying that ASA-IgG2 molecules remain monomeric without the addition of NaCl. This is in close agreement with *k_D_* and *S(q)_eff_* results, as they all suggest the net PPI were repulsive under equivalent conditions (Figure 2). Figure 6 also shows the molar ratios of various oligomeric species in 300 mM, 600 mM, and 1200 mM NaCl solutions. For these samples, the molar fraction of monomer decreased significantly with a concomitant increase in the number of multimers, including dimers, trimers, and hexamers. As discussed earlier, *S*(0)*_eff_* values measured from 50 mg/mL solution became greater than 1 when *C_NaCl_* increased to ≥300 mM, implying that the net PPI were dominated by attractive interactions; therefore, the CG-MALS results also confirmed the formation of higher-order structures. The observed conversion of monomers to higher-order oligomers through CG-MALS experiments suggested the presence of NaCl in solution led to an increase in the association constant of ASA-IgG2 molecules.

### 3.3. Empirical Relationship between k_D_, B_*22*_, S(q)_eff_ and Solution Viscosity

Both *k_D_* and *B*_22_ are widely used to characterize PPI, yet they are measured at low mAb concentrations, whereas increases in viscosity largely occur at much higher concentrations. Fundamentally, the role of PPI and viscosity of concentrated mAb formulations is influenced by the particular atomic-level detail in terms of amino acid sequence, post-translational modifications, and the internal flexibility of mAb molecules. This is clearly evident in cases where a single or a small number of mutations of mAb molecules lead to dramatic changes in viscosity [74,75,76]. Creating models and simulations to include this level of detail is an area of active research [10,77,78,79]. Validation of molecular dynamics trajectories requires the use of experimental measurements that reproduce the solution properties at multiple phase points. In addition, the validity of extrapolating the properties inherent in *k_D_* and *B*_22,_ obtained at low concentrations to high concentrations and, therefore, the resultant effect on solution viscosity can be evaluated directly by evaluating model interaction parameters obtained via *S(q)_eff_*, which is measured at both low and high concentrations.

**Figure 7:**
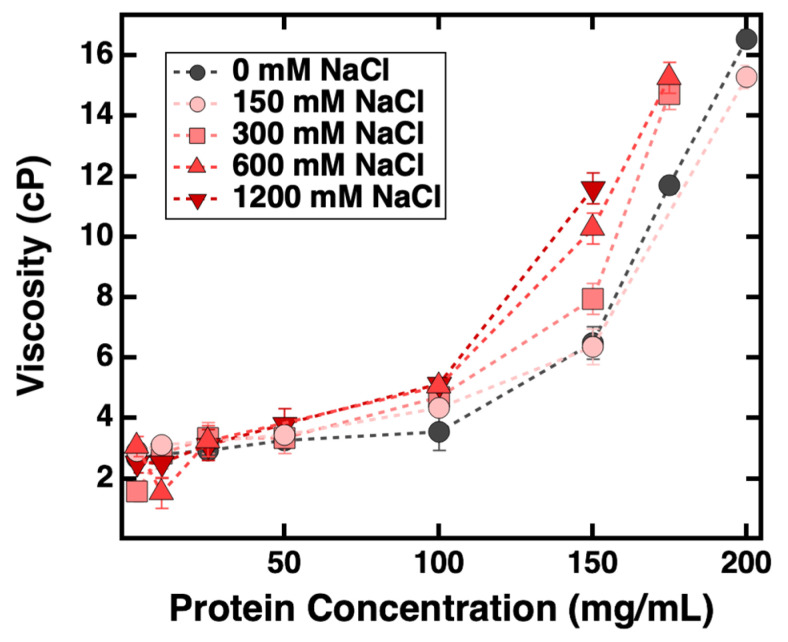
Viscosity of ASA-IgG2 solutions prepared at various mAb and NaCl concentrations. Dotted lines are included to guide the eye. Error bars represent one standard deviation from three separate measurements.

Thus, the focus of the current study is to evaluate whether an empirical relationship exists between the experimentally determined viscosity results and those predicted from interaction parameters used in colloid models (derived from *k_D_, B*_22_ and *S(q)_eff_*). Figure 7 shows that changes in viscosity followed the same trend for samples prepared with all five different NaCl concentrations, in that significant increases in solution viscosity were observed as *C_mAb_* reached 150 mg/mL. In contrast, *C_NaCl_* did not influence the measured viscosity when *C_mAb_* was below 150 mg/mL.

It is commonly accepted that solution viscosity is expected to increase under conditions where net PPI are attractive, and the viscosity should remain low where net PPI are repulsive [40]. Since *k_D_* and *B*_22_ were measured from dilute protein solutions, there was only one *k_D_* and *B*_22_ value measured from each *C_NaCl_*. In this study, both *k_D_* and *B*_22_ values suggest the net PPI were repulsive among all examined NaCl concentrations; therefore, it was expected that the viscosity of mAb solutions should remain low with increasing *C_mAb_*. However, the experimentally determined viscosity results showed that the viscosity of mAb solutions elevated significantly with increasing *C_mAb_*. Therefore, the predicted viscosity based on properties measured from dilute solutions did not agree with experimental data, suggesting *k_D_* and *B*_22_ are not ideal parameters for predicting viscosity at high *C_mAb_*. Since the viscosity of ASA-IgG2 solutions demonstrated dependence on both *C_mAb_* and *C_NaCl_*, we examined if there was an empirical relationship between *S(q)_eff_* and the solution viscosity measured from each *C_mAb_* and *C_NaCl_* combination.

**Figure 8:**
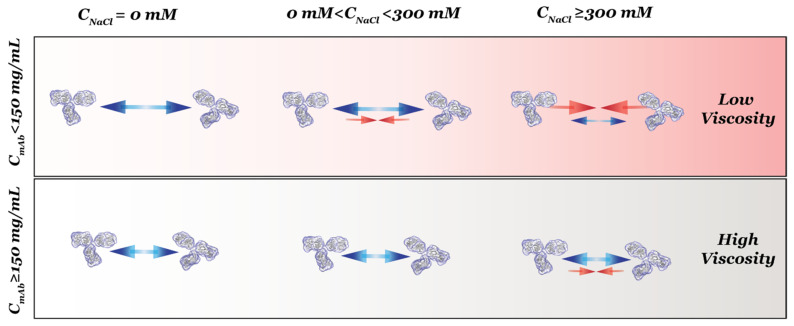
Illustration of PPI among ASA-IgG2 under conditions varying in mAb and NaCl concentrations. The two rows represent samples prepared with *C_mAb_* either below or above a critical concentration of 150 mg/mL, while the columns are divided into three *C_NaCl_* regimes. Blue and red arrows represent repulsive and attractive inter-protein interactions, respectively. Dominating interactions are illustrated with bigger arrows, whereas non-dominating interactions are illustrated with smaller arrows.

As summarized in Figure 8, the effects of NaCl on solution viscosity were not significant when *C_mAb_* was below 150 mg/mL. In this regime, although the net PPI changed from repulsive to attractive with increasing *C_NaCl_* (as evident by *S(q)_eff_* values), solution viscosity remained low. For samples prepared with a *C_mAb_* greater than 150 mg/mL, viscosity measurements demonstrated that the viscosity of mAb solutions increased with the emergence of attractive forces despite the PPI being net repulsive. Therefore, in the case of ASA-IgG2, the overall attractive PPI do not necessarily lead to increased solution viscosity, and the overall repulsive PPI can be observed in solutions with elevated viscosity. Thus, the observed empirical relationship between the measured and predicted viscosity results in this study contradicts the commonly accepted relationship between net PPI and the viscosity of concentrated mAb formulations. Similar results were reported by Woldeyes et al., who examined whether experimentally determined viscosity results agreed with those predicted from PPI measured from both dilute and concentrated mAb formulations [43]. They performed DLS/SLS and viscosity experiments to determine the viscosity of four different mAbs when prepared in solutions with and without 150 mM NaCl. Their study did not include high *C_NaCl_* measurements, and no SAXS/SANS or CG-MALS was performed to characterize the PPI among concentrated mAbs. Without the direct observation of interactions via *S(q)_eff_* and oligomeric content, they postulated that PPI could not be used to provide reliable predictions on the viscosity of mAb formulations, no matter if the PPI were measured from dilute or concentrated samples [43]. It is anticipated that the nature and significance of various PPI can change dramatically when mAb molecules are at higher concentrations, with concomitant reduced intermolecular distances and, therefore, an increased propensity of intermolecular association. To obtain reliable viscosity predictions, further studies are needed to establish the relationship between the measured viscosity and PPI measured from concentrated mAb formulations.

## 4. Conclusions

In this study, two sets of interaction parameters were obtained to characterize the stability and viscosity of ASA-IgG2 formulations with modulation from NaCl. They were *k_D_*/*B*_22_ measured from dilute samples and *S(q)_eff_* measured from concentrated samples. Our results showed that the nature of net PPI changed not only with *C_NaCl_* but also with increasing *C_mAb_*. As a result, interaction parameters measured from dilute and concentrated mAb samples could lead to different predictions on the stability of mAb formulations. We also showed that the viscosity of mAb solutions could not be accurately predicted using PPI based on our current knowledge of the relationship between PPI and solution viscosity. However, it is not only PPI that can be used to explain the viscosity of mAb solutions. Here, we also looked at the viscosity of protein solutions from the point of simple chemical equilibrium. As mentioned earlier, the monomeric mAb molecules are in constant equilibrium with higher-order oligomers, including dimers, trimers, hexamers, etc., as evident from our CG-MALS results. Previous studies also demonstrate that the association constant *K* of various mAbs can be in the micromolar to millimolar range [20,80]. Therefore, reversible oligomers are always present in mAb solutions. An increase in mAb concentration will promote self-association of monomers, and the extent of oligomer formation is controlled by the association constant *K*. As an initial approximation, with an increased number of higher-order oligomers that are bigger in size, the viscosity of mAb solutions will rise, and it is expected that the change in viscosity should be related to the distribution of oligomeric species. Note that there was no evidence in the scattering data for long-length scale networks of mAb molecules; thus, the spatial and temporal factors of the population of monomeric and oligomeric species on the viscosity behavior remains an open question. Therefore, the complete mechanism of the self-association propensity of mAbs to improve the prediction of bulk viscosity in concentrated formulations will rely on measurements carried out at high concentrations to account for the correct physics in the phase points of interest.

## Data Availability

The data presented in this study are available in the article and supporting information.

## Supplementary Materials

The following supporting information can be downloaded at: https://www.mdpi.com/article/10.3390/antib11020024/s1, Figure S1: The closer look at the scattering profiles measured from 50 mM NaCl solutions around the *q*-range from 0.10 Å^−1^ to 0.14 Å^−1^. This linear region from various scattering profiles should be well overlapped if the concentration normalization is done properly. Error bars in the scattering profiles represent the relative uncertainties in the scattering intensity measurements based on counting statistics. Figure S2: Summary of SAXS profiles measured from ASA-IgG2 samples prepared in various NaCl solutions: 0 mM NaCl (**A**), 50 mM NaCl (**B**), 100 mM NaCl (**C**), and 150 mM NaCl (**D**). The scattering profiles measured from various mAb concentrations were concentration-normalized. In particular, *I(q)* measured from 2 mg/mL and 10 mg/mL solutions looked identical, this implied that the increase of protein concentration from 2 mg/mL to 10 mg/mL did not change the total scattering profiles, thus *S(q)_eff_* were absent from both concentrations. A decrease in *I(q)* at low-*q* region started to appear at 25 mg/mL and it became more significant with increasing protein concentrations. The observed reduction in scattering intensity at low-q region was due to the presence of intermolecular interactions; therefore, *S(q)_eff_* started to arise when the mAb concentration reached 25 mg/mL for 50 mM NaCl formulation. Error bars in the scattering profiles represent the relative uncertainties in the scattering intensity measurements based on counting statistics. Figure S3: Summary of SANS profiles measured from ASA-IgG2 samples prepared in various NaCl solutions: 0 mM NaCl (**A**), 300 mM NaCl (**B**), 600 mM NaCl (**C**), and 1200 mM NaCl (**D**). The scattering profiles measured from various mAb concentrations were concentration-normalized. As mentioned in the method section, samples with high mAb and NaCl concentrations could not be made due to difficulties in preparing concentrated ASA-IgG2 (greater than 215 mg/mL) and NaCl (greater than 5 M) stock solutions. Error bars in the scattering profiles represent the relative uncertainties in the scattering intensity measurements based on counting statistics. Figure S4: Summary of *S(q)_eff_* profiles measured from ASA-IgG2 samples prepared in various NaCl solutions: 0 mM NaCl (**A**), 50 mM NaCl (**B**), 100 mM NaCl (**C**), 150 mM NaCl (**D**), 300 mM NaCl (**E**), 600 mM NaCl (**F**), and 1200 mM NaCl (**G**). *S(q)_eff_* profiles were fitted using with Hayter-Penfold and Two-Yukawa models to extrapolate *S(q)_eff_* values. Error bars in the scattering profiles represent the relative uncertainties in the scattering intensity measurements based on counting statistics.
