# Supplementary material for: Effects of Monovalent Salt on Protein-Protein Interactions of Dilute and Concentrated Monoclonal Antibody Formulations
Source: Antibodies (Basel). 2022 Mar 31;11(2):24. doi: 10.3390/antib11020024 (PMC9036246; doi:10.3390/antib11020024)
Supplement: Supplementary file 1 [file antibodies-11-00024-s001.zip › antibodies-1616017-supplementary_AX.pdf]

Effects of Monovalent Salt on Protein-Protein Interactions of Dilute and Concentrated Monoclonal Antibody Formulations

Amy Y. Xu [1,2,†], Nicholas J. Clark [1,3], Joseph Pollastrini [3], Maribel Espinoza [3], Hyo-Jin Kim [3], Sekhar Kanapuram [3], Bruce Kerwin [3], Michael J. Treuheit [3], Susan Krueger [1], Arnold McAuley [3] and Joseph E. Curtis [1]

[1] NIST Center for Neutron Research, National Institute of Standards and Technology, 100 Bureau Drive, Mail Stop 6102, Gaithersburg, Maryland 20899, United States
[2] Institute for Bioscience and Biotechnology Research, University of Maryland, 9600 Gudelsky Drive, Rockville, Maryland 20850, United States
[3] Amgen Inc., One Amgen Center Drive, Thousand Oaks, California 91320, United States
† Present Address: Department of Chemistry, Louisiana State University, Baton Rouge, Louisiana.

Theoretical Background for $S(q)_{eff}$ analysis

The theoretical background for $S(q)_{eff}$ analysis presented in our study is provided in one of our previous publications [1]. For readers' convenience, it is restated below.

Historical integral-equation theories are generally based on isotropic potentials of spherically symmetric particles [2]. Under the assumption that particle size and orientation are not correlated, the scattering intensity $I(q)$ as a function of momentum transfer q can be written as:

$$I(q) = n_p P(q) S(q)_{eff} \tag{S1}$$

where n_p is the number density of particles in the sample, $P(q)$ is the single-particle form factor, and $S(q)_{eff}$ is the effective structure factor. The magnitude of momentum transfer q can be related to the length scale d, using:

$$d = \frac{2\pi}{q} \tag{S2}$$

$P(q)$ can be calculated from the complex scattering amplitudes $F(q)$ as follows:

$$P(q) = \langle |F(q)|^2 \rangle \tag{S3}$$

where the brackets represent both angular and ensemble averages. $F(q)$ can be calculated directly from molecular simulations using a simple Debye sum for N particles [3], each $j(k)$ particle with scattering length $b_j(b_k)$ and atomic position $X_j(X_k)$ using:

$$F(q) = \sum_{k=1}^{N} \sum_{j=1}^{N} b_j b_k \exp[iq(X_j - X_k)] \tag{S4}$$

The decoupling approximation has been proposed to calculate the structure factor $S(q)$ from $S(q)_{eff}$ using [4]:

$$S(q)_{eff} = 1 + \beta(q)[S(q) - 1] \tag{S5}$$

where the decoupling factor $\beta(q)$ is defined as[4]:

$$\beta(q) = \frac{|\langle F(q) \rangle|^2}{\langle |F(q)|^2 \rangle} \tag{S6}$$

The value of $\beta(q)$ varies between 0 and 1, and it can serve to decouple molecular shape from intermolecular particle interactions. $S(q)$ is the Fourier transform of the pair distribution function $g(r)$, which is the probability of finding one particle at a distance r from another particle in the system. $S(q)$ can be written in terms of $g(r)$ as:

$$S(q) = 1 + 4\pi n_p \int_0^\infty (g(r) - 1) \frac{\sin(qr)}{qr} r^2 dr \tag{S7}$$

where $g(r)$ depends on the interaction potential, since molecules have higher probabilities to be found at distances corresponding to lower interaction energies. Moreover, for a given interaction potential, Equation (S7) can be solved using the Ornstein-Zernike equation and an appropriate closure relation [5,6]. Therefore, obtaining $S(q)$ from experimental data is useful to evaluate the net interactions governing the system. Besides the decoupling approximation, other approaches have been proposed to account for shape anisotropy in $S(q)_{eff}$, such as assuming a sphere with an effective diameter that matches the second virial coefficient of the molecule [7–10].

In one of our previous studies, we showed that inter-protein interactions assessed from $S(q)_{eff}$ are not perturbed by structural changes in mAb for $q < 0.02$ Å^{-1}. In this study, we used $S(0)_{eff}$ values to determine the nature of net PPI. Since $S(0)_{eff}$ values were extrapolated from $S(q)_{eff}$ when q is approaching zero (i.e. less than q = 0.02 Å^{-1}), for this purpose, $S(q)_{eff}$ profiles can be fitted using statistical mechanical models of the $S(q)$ and consider mAbs as isotropic spheres.

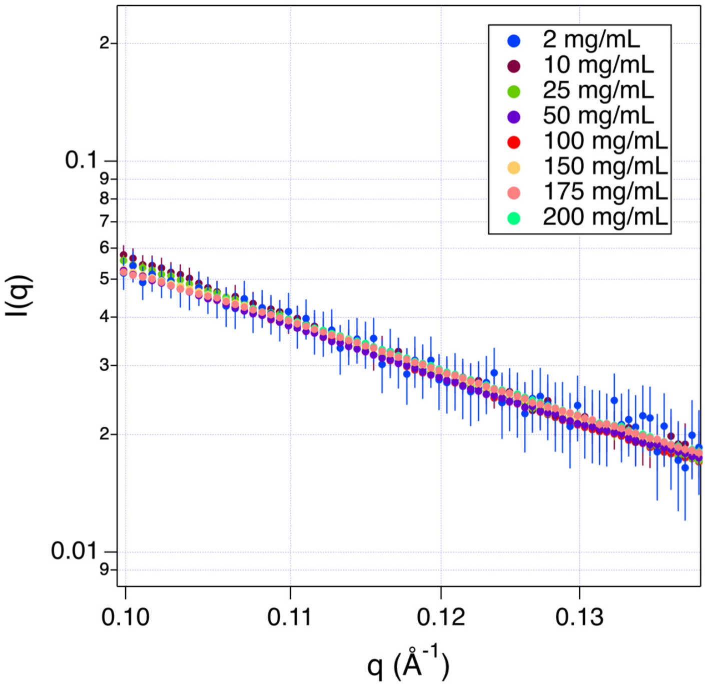

Figure S1. The closer look at the scattering profiles measured from 50 mM NaCl solutions around the q-range from 0.10 Å^{-1} to 0.14 Å^{-1}. This linear region from various scattering profiles should be well overlapped if the concentration normalization is done properly. Error bars in the scattering profiles represent the relative uncertainties in the scattering intensity measurements based on counting statistics.

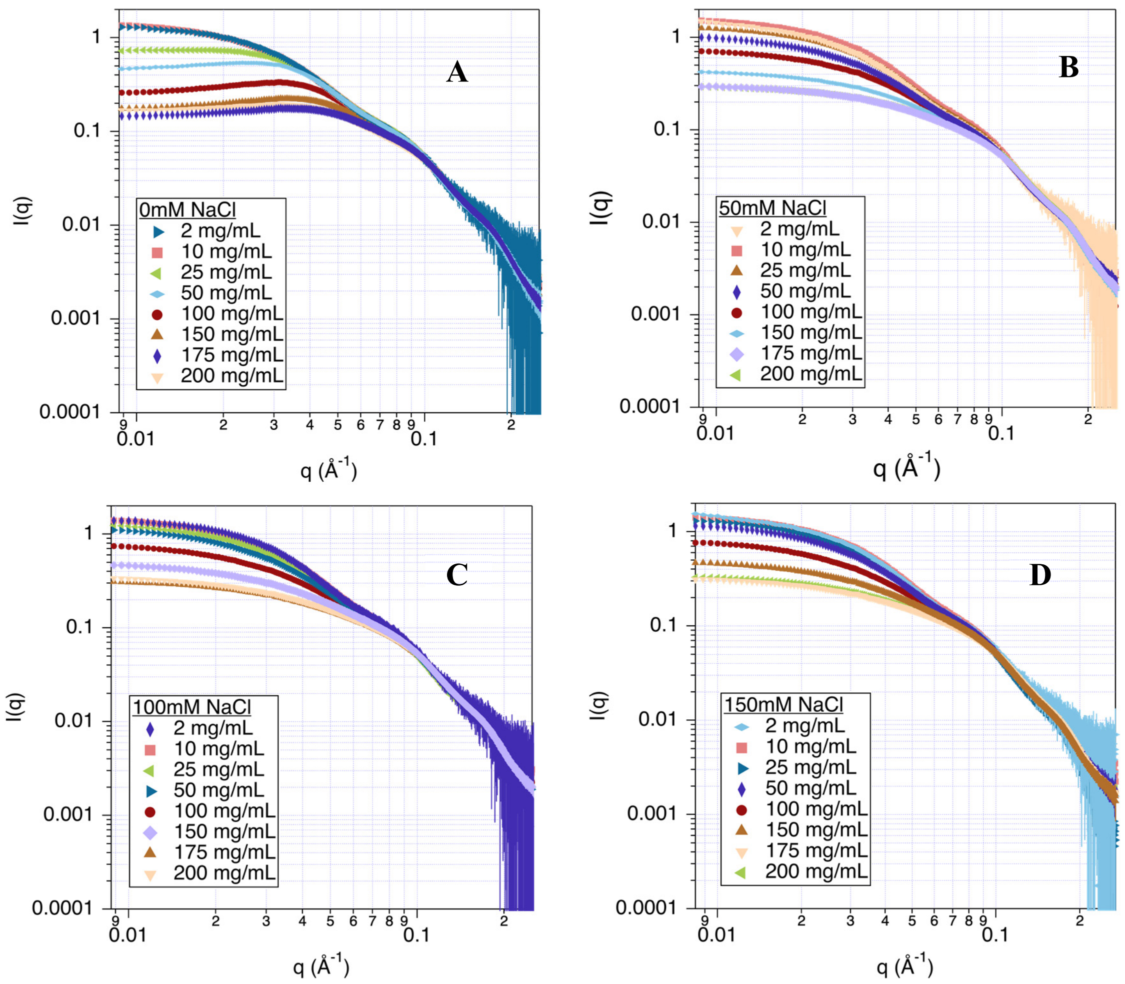

Figure S2. Summary of SAXS profiles measured from ASA-IgG2 samples prepared in various NaCl solutions: 0 mM NaCl (**A**), 50 mM NaCl (**B**), 100 mM NaCl (**C**), and 150 mM NaCl (**D**). The scattering profiles measured from various mAb concentrations were concentration-normalized. In particular, *I(q)* measured from 2 mg/mL and 10 mg/mL solutions looked identical, this implied that the increase of protein concentration from 2 mg/mL to 10 mg/mL did not change the total scattering profiles, thus $S(q)_{eff}$ were absent from both concentrations. A decrease in *I(q)* at low-*q* region started to appear at 25 mg/mL and it became more significant with increasing protein concentrations. The observed reduction in scattering intensity at low-q region was due to the presence of intermolecular interactions; therefore, $S(q)_{eff}$ started to arise when the mAb concentration reached 25 mg/mL for 50 mM NaCl formulation. Error bars in the scattering profiles represent the relative uncertainties in the scattering intensity measurements based on counting statistics.

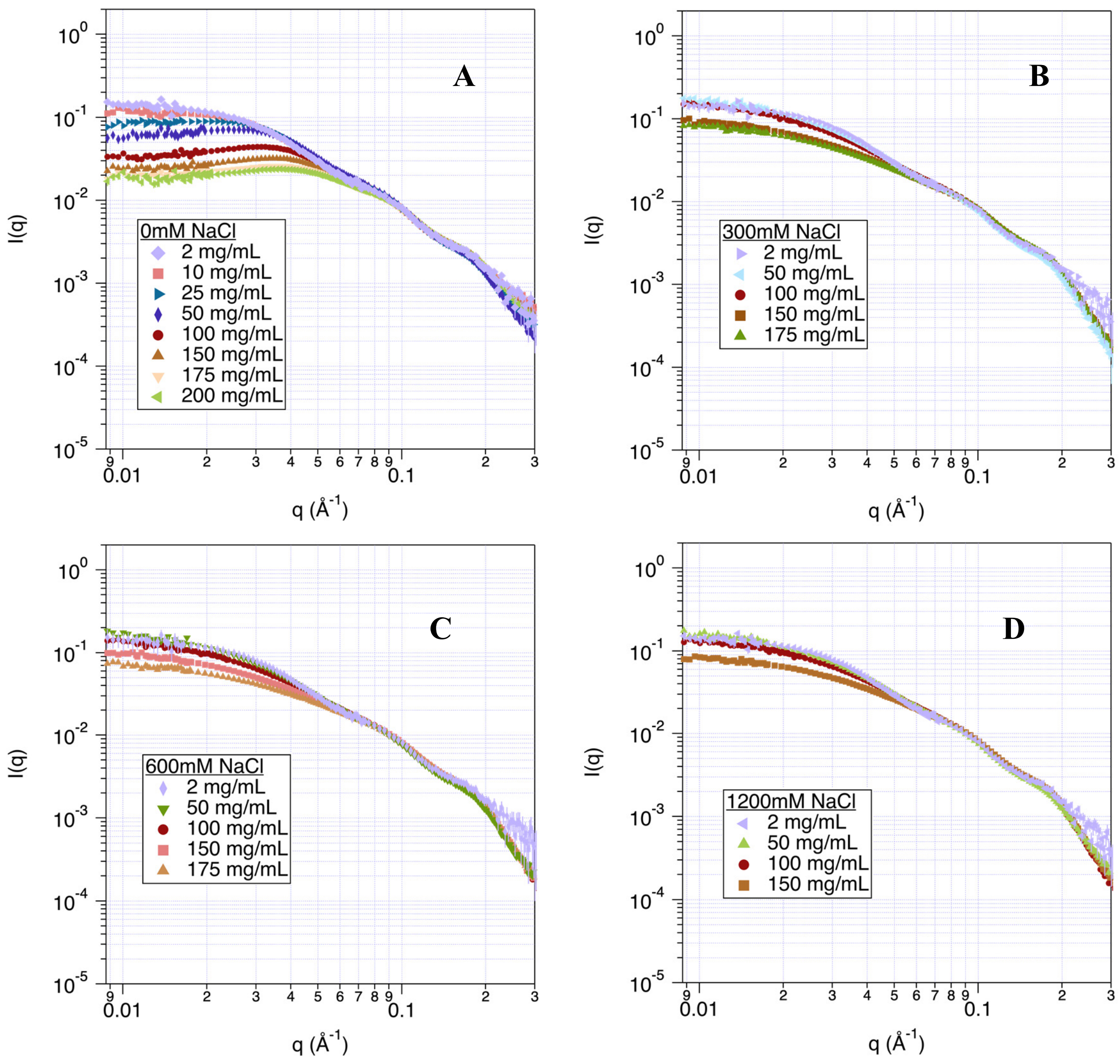

Figure S3. Summary of SANS profiles measured from ASA-IgG2 samples prepared in various NaCl solutions: 0 mM NaCl (**A**), 300 mM NaCl (**B**), 600 mM NaCl (**C**), and 1200 mM NaCl (**D**). The scattering profiles measured from various mAb concentrations were concentration-normalized. As mentioned in the method section, samples with high mAb and NaCl concentrations could not be made due to difficulties in preparing concentrated ASA-IgG2 (greater than 215 mg/mL) and NaCl (greater than 5 M) stock solutions. Error bars in the scattering profiles represent the relative uncertainties in the scattering intensity measurements based on counting statistics.

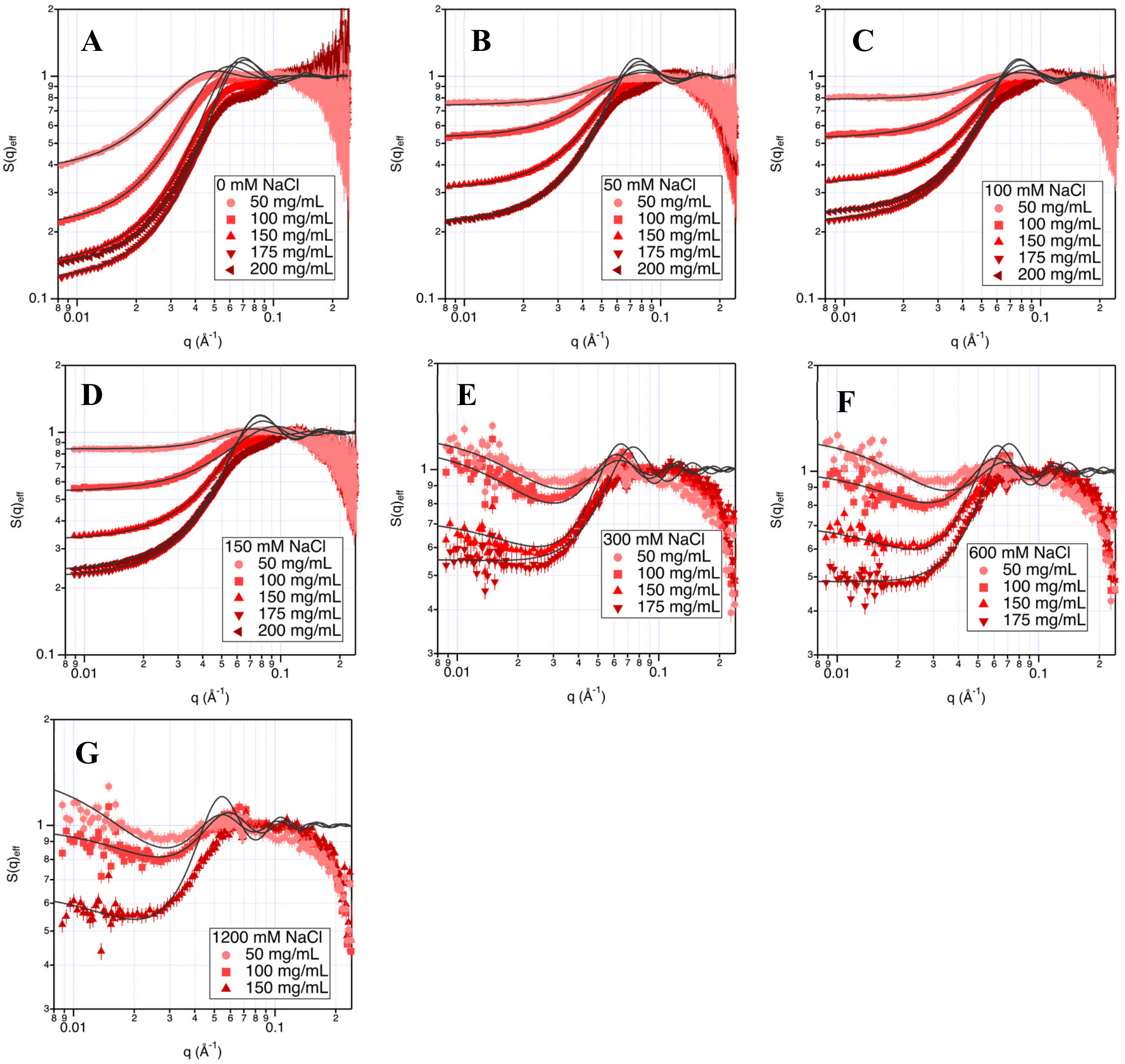

Figure S4. Summary of $S(q)_{eff}$ profiles measured from ASA-IgG2 samples prepared in various NaCl solutions: 0 mM NaCl (**A**), 50 mM NaCl (**B**), 100 mM NaCl (**C**), 150 mM NaCl (**D**), 300 mM NaCl (**E**), 600 mM NaCl (**F**), and 1200 mM NaCl (**G**). $S(q)_{eff}$ profiles were fitted using with Hayter-Penfold and Two-Yukawa models to extrapolate $S(q)_{eff}$ values. Error bars in the scattering profiles represent the relative uncertainties in the scattering intensity measurements based on counting statistics.

References

1. Castellanos, M.M.; Clark, N.J.; Watson, M.C.; Krueger, S.; McAuley, A.; Curtis, J.E. Role of Molecular Flexibility and Colloidal Descriptions of Proteins in Crowded Environments from Small-Angle Scattering. *J. Phys. Chem. B* **2016**, *120*, 12511–12518.
2. McQuarrie, D.A. *Quantum chemistry*. University Science Books: Sausalito, CA, USA, 2008. ISBN-13:978-18913895043.
3. Debye, P. Zerstreuung von röntgenstrahlen. *Annalen der Physik* **1915**, *351*, 809–823.
4. Kotlarchyk, M.; Chen, S.H. Analysis of small angle neutron scattering spectra from polydisperse interacting colloids. *J. Phys. Chem.* **1983**, *79*, 2461–2469.
5. Hayter, J.B.; Penfold, J. An analytic structure factor for macroion solutions. *Mol. Phys. 1981, 42, 109–118.*
6. Percus, J.K.; Yevick, G.J. Analysis of classical statistical mechanics by means of collective coordinates. *Phys. Rev.* **1958**, *110*, 1.
7. Baravian, C.; Michot, L.; Paineau, E.; Bihannic, I.; Davidson, P.; Impéror-Clerc, M.; Belamie, E.; Levitz, P. An effective geometrical approach to the structure of colloidal suspensions of very anisometric particles. *EPL* **2010**, *90*, 36005.
8. Hansen, S. Monte Carlo estimation of the structure factor for hard bodies in small-angle scattering. *J. Appl. Crystallogr.* **2012**, *45*, 381–388.
9. Kline, S.R. Reduction and analysis of SANS and USANS data using IGOR Pro. *J. Appl. Crystallogr.* **2006**, *39*, 895–900.
10. Zhang, F.; Skoda, M.W.; Jacobs, R.M.; Martin, R.A.; Martin, C.M.; Schreiber, F. Protein interactions studied by SAXS: effect of ionic strength and protein concentration for BSA in aqueous solutions. *J. Phys. Chem. B* **2007**, *111*, 251–259.
